# Sorafenib Inhibits Lymphoma Xenografts by Targeting MAPK/ERK and AKT Pathways in Tumor and Vascular Cells

**DOI:** 10.1371/journal.pone.0061603

**Published:** 2013-04-19

**Authors:** Carmelo Carlo-Stella, Silvia L. Locatelli, Arianna Giacomini, Loredana Cleris, Elena Saba, Marco Righi, Anna Guidetti, Alessandro M. Gianni

**Affiliations:** 1 Department of Oncology and Hematology, Humanitas Cancer Center, Humanitas Clinical and Research Center, Rozzano, Milano, Italy; 2 Department of Medical Biotechnology and Translational Medicine, University of Milano, Milano, Italy; 3 Experimental Oncology, Fondazione IRCCS Istituto Nazionale Tumori, Milano, Italy; 4 National Research Council, Institute of Neuroscience, Milan, Italy; 5 Medical Oncology, Fondazione IRCCS Istituto Nazionale Tumori, Milano, Italy; 6 Department of Medical Physiopathology and Transplants, University of Milano, Milano, Italy; European Institute of Oncology, Italy

## Abstract

The anti-lymphoma activity and mechanism(s) of action of the multikinase inhibitor sorafenib were investigated using a panel of lymphoma cell lines, including SU-DHL-4V, Granta-519, HD-MyZ, and KMS-11 cell lines. *In vitro*, sorafenib significantly decreased cell proliferation and phosphorylation levels of MAPK and PI3K/Akt pathways while increased apoptotic cell death. *In vivo*, sorafenib treatment resulted in a cytostatic rather than cytotoxic effect on tumor cell growth associated with a limited inhibition of tumor volumes. However, sorafenib induced an average 50% reduction of tumor vessel density and a 2-fold increase of necrotic areas. Upon sorafenib treatment, endothelial and tumor cells from SU-DHL-4V, Granta-519, and KMS-11 nodules showed a potent inhibition of either phospho-ERK or phospho-AKT, whereas a concomitant inhibition of phospho-ERK and phospho-AKT was only observed in HD-MyZ nodules. In conclusion, sorafenib affects the growth of lymphoid cell lines by triggering antiangiogenic mechanism(s) and directly targeting tumor cells.

## Introduction

Sorafenib is an oral multitargeted inhibitor of kinases approved by the Food and Drug Administration for the treatment of patients with advanced renal cell carcinoma (RCC) and those with unresectable hepatocellular carcinoma (HCC) [1]–[3]. It is also approved by the European Medicines Agency for the treatment of patients with HCC and patients with advanced RCC with whom prior interferon-α- or interleukin-2-based therapy had failed or those considered to be unsuitable for such therapy. Sorafenib is currently undergoing phase II/III clinical evaluation in a wide variety of other solid as well as hematopoietic tumors [4]–[7].

In addition to targeting the receptor tyrosine kinases c-kit, Flt3, and RET [8], [9], sorafenib at a clinically achievable plasma concentration of 10 µM, inhibits multiple additional receptor tyrosine kinases and intracellular kinases [10]. By inhibiting the Raf/mitogen-activated protein (MAP)/extracellular-signal regulated kinase (ERK) kinase (MEK)/ERK (Raf/MEK/ERK) signaling pathway sorafenib exerts direct antiproliferative, apoptotic and antiangiogenic effects on a variety of solid tumors as well as leukemic cell lines [11]. The antiproliferative activity of sorafenib varies from nanomolar to micromolar concentrations depending on the oncogenic signaling pathways driving proliferation. The proapoptotic effects of sorafenib may vary among cell lines and result from mechanism(s) of action that are only partially elucidated. A commonly observed feature is the inhibition of phosphorylation of the initiation factor eIF4E and downregulation of the antiapoptotic protein myeloid cell leukemia-1 (Mcl-1) [12], [13], a Bcl-2 family member implicated in malignant hematopoietic cell survival. Downregulation of Mcl-1 by sorafenib is associated with the release of cytochrome c from mitochondria into the cytosol, caspase activation, and apoptotic cell death [14]. The ability of sorafenib to suppress tumor angiogenesis has been well established and is likely due to the potent inhibition of the proangiogenic vascular endothelial growth factor receptor (VEGFR1)-1, VEGFR-2, VEGFR-3, and platelet-derived growth factor receptor-ß [9]. In vivo, sorafenib exhibits antitumor activity against a variety of human xenograft models of multiple histological types, including melanoma, renal, colon, pancreatic, hepatocellular, lung, and thyroid carcinomas, as well as acute myelogenous leukemia [7], [15]–[17]. Furthermore, sorafenib produced partial tumor regressions in mice bearing PLC/PRF/5 HCC and induced tumor regression in a breast cancer model harboring B-Raf and K-Ras oncogenic mutations [18].

Several lines of in vitro evidence suggest that sorafenib might have a role in the treatment of lymphomas by overcoming the cytoprotective effects of ERK, and Mcl-1 and eventually targeting oncogenic signaling pathways driving lymphomagenesis [19]–[23]. Indeed, sorafenib-induced inhibition of the ERK1/2 pathway might results in Bcl-X_L_ downregulation [24], thus mimicking rituximab-mediated effects on CD20-positive non-Hodgkin lymphoma cell lines [25]. Additionally, sorafenib might downregulate Mcl-1 [14], which is implicated in resistance to anticancer drugs and is overexpressed in a significant proportion of diffuse large B cell lymphoma (DLBCL) and follicular lymphoma (FL) [26], thereby restoring lymphoma cell sensitivity to apoptosis.

It was the aim of the present study to further investigate in preclinical models both in vitro and in vivo the antitumor activity and mechanism(s) of action of sorafenib using a panel of cell lines representative of the heterogeneity of lymphoproliferative disorders. Data reported herein demonstrate that pharmacologically achievable concentrations of sorafenib inhibit the growth of lymphoid cell lines of different histotypes, and suggest that both antiangiogenic mechanism(s) as well as direct targeting of tumor cells mediate the in vivo antilymphoma activity of sorafenib.

## Materials and Methods

### Reagents

Sorafenib (Bayer AG, Leverkusen, Germany, EU) was dissolved in 100% dimethylsulfoxide (DMSO) to obtain a stock solution (60 mg/ml). Working solutions (100×) were obtained by further diluting in RPMI-1640 and were added to in vitro cultures at 1% (v/v) to achieve a final DMSO concentration of 0.01%.

### Cell lines and primary cells

SU-DHL-4V (DLBCL), Granta-519 [mantle cell lymphoma (MCL)], HD-MyZ [Hodgkin lymphoma (HL)], and KMS-11 [multiple myeloma (MM)] cell lines were purchased from the German Collection of Microorganisms and Cell Cultures (DSMZ, Braunschweig, Germany, EU). Cell lines were cultured in RPMI-1640 supplemented with 10% fetal bovine serum (FBS) and periodically tested for mycoplasma contamination. Primary DLBCL and MCL mononuclear cells (MNCs) were isolated at the time of diagnostic work-up from the peripheral blood of consenting patients. As assessed by flow cytometry, percentages of neoplastic cells were always >95%.

### Apoptosis and viable cell counting

Apoptotic cells were detected by annexin-V/propidium iodide (PI) double staining and flow cytometry analysis, as previously described [27]. Viable cells, i.e. annexin-V^−^/PI^−^ cells, were counted with Flow-Count beads (Beckman Coulter, Milano, Italy, EU) by flow cytometry. The proportion of viable cells was further determined by an WST-based vitality assay following the manufacturer's recommendations (Biovision, Milpitas, CA, USA).

### Measurement of ΔΨm

Mitochondrial membrane depolarization was determined by using the fluorescent probe TMRE and flow cytometry, as previously described [28]. The fluorescent dye TMRE is accumulated by mitochondria and as a results of mitochondrial membrane depolarization, a shift to the left in the emission spectrum by apoptotic cells can be detected.

### Western blot analysis

Cell samples were homogenized in NP-40 lysis buffer (1% NP-40, 20 mM Tris-HCl pH 8, 137 mM NaCl, 10% glycerol, 2 mM EDTA, 1 mM sodium orthovanadate, 10 µg/mL aprotinin, 10 µg/mL leupeptin). Cell lysates were resolved by electrophoresis on a 10% polyacrylamide gel and transferred to nitrocellulose membranes. Immunocomplexes were visualized using an enhanced chemiluminescence Western blotting detection system (Amersham Biosciences, Milano, Italy, EU). Blotting analysis was performed using anti-Mcl-1, anti-phospho-MEK, -ERK,-AKT S473, -S6, GSK-3 α/β, anti-p38α, -caspase-8 antibodies from Cell Signaling (Danvers, MA, USA), anti-caspase-3 antibody (Santa Cruz, San Diego, CA, USA), anti-caspase-9 and anti-poly(ADP-ribose)polymerase (PARP) (B–D).

### Phospho-MAPK proteome profiler array

Untreated and sorafenib-treated cells were washed with ice-cold PBS and solubilized in NP-40 lysis buffer (1% NP-40, 20 mM Tris-HCl pH 8, 137 mM NaCl, 10% glycerol, 2 mM EDTA, 1 mM sodium orthovanadate, 10 µg/mL aprotinin, 10 µg/mL leupeptin). Cell lysates were centrifuged (14,000 rpm, 5 minutes, 4°C), and protein concentrations in the supernatants was determined using the Bradford assay (Bio-Rad Laboratories). Equal amounts of proteins (200 µg) from control and sorafenib-treated cells were incubated with the human phospho-MAPK array kit. The following kinases were detected: ERK1 (T202/Y204), ERK2 (T185/Y187), c-Jun N-terminal kinase (JNK) pan (T183/Y185, T221/Y223), p38α (T180/Y182), glycogen synthase kinase (GSK)-3 α/β (S21/S9), GSK-3 β (S9), Akt pan (S473, S474, S472), Akt1 (S473), Akt2 (S474), Akt3 (S472), p70 S6K (T421/S424), and HSP27 (S78/S82). Phospho-MAPK array data developed on X-ray films (Amersham Biosciences) following exposure to chemiluminescent reagents were analyzed using the open source imaging software ImageJ (http://rsb.info.nih.gov/ij/). The assays were conducted in duplicate. A ratio of signal intensity (sorafenib∶control) was calculated for each of the four replicates (two duplicates per assay) and transformed into a log value (base 10).

### *In vivo* activity of sorafenib in tumor-bearing non-obese diabetic/severe combined immunodeficient (NOD/SCID) mice

Six- to eight-week-old NOD/SCID mice were purchased from Charles River (Milano, Italy, EU) and xenografted with SU-DHL-4V, Granta-519, HD-MyZ, or KMS-11 cell lines [27]. Mice were housed under standard laboratory conditions according to our institutional guidelines. Animal experiments were performed according to the Italian laws (D.L. 116/92 and following additions), which enforce the EU 86/109 Directive, and were approved by the institutional Ethical Committee for Animal Experimentation of the National Cancer Institute Foundation. Tumor cells (5×10^6^ cells/mouse) were inoculated subcutaneously (SC) in the right flank of each mouse. When tumor volume reached approximately 100 mg in weight (6–13 days after tumor inoculation), mice were randomly assigned to receive either a short- or long-term treatment with sorafenib diluted in DMSO (final concentration, 10% v/v) or control vehicle (DMSO 10%, v/v). In preliminary experiments, mice injection with a 10% DMSO solution failed to affect tumor cell signaling likely due to an in vivo DMSO dilution as well as metabolism. The short-term treatment consisting of intraperitoneal (IP) sorafenib (90 mg/kg) or DMSO for 5 days was used to assess necrotic areas and tumor vascularity. The long-term treatment consisted of sorafenib (90 mg/kg) or DMSO 5 days per week for 3 weeks. The endpoint of the long-term treatment was tumor weight. The tumors were measured with calipers, and their weights were calculated using the formula: (a×b^2^)/2, where a and b represented the longest and shortest diameters, respectively. Antitumor efficacy was measured as tumor growth inhibition (TGI) defined as [1−(T/C)×100], where T and C are the mean tumor weight in the treated and untreated control groups, respectively. Mice were monitored twice weekly and were killed by cervical dislocation when they showed signs of terminal illness, including hind leg paralysis, inability to eat or drink, and/or moribund. Each experiment was performed on at least two separate occasions, using five mice per experiment.

### Analysis of tumor nodules

Tumor vasculature was analyzed by in vivo staining using sulfosuccinimidyl-6-(biotinamido) hexanoate (sulfo-NHS-LC-biotin, Thermo Fisher Scientific, Rockford, IL, USA) [27], [29]. Biotinylated tumors were snap-frozen in isopentane in liquid nitrogen. Tumor endothelial cells were then revealed by immunohistochemistry using HRP-conjugated streptavidin (Dako, Milano, Italy, EU) or immunofluorescence using Alexa Fluor 488-conjugated streptavidin (Invitrogen, Milano, Italy, EU). Formalin-fixed, paraffin-embedded tumor nodules were stained with hematoxylin and eosin (H&E) or processed for immunohistochemistry with anti-mouse CD31 (Santa Cruz Biotechnology, Inc., Heidelberg, Germany, EU) and anti-human Ki-67 (Dako). Tumor necrosis was detected using TdT-mediated dUTP nick end-labeling (TUNEL) staining (Roche, Milano, Italy, EU) according to the manufacturer's instructions. Positive signal was revealed by 3,3-diaminibenzidine staining, and tumor sections were then counterstained before analysis by light microscopy.

### Analysis of stained sections

Entire tissue sections were acquired at 20× magnification with an automatic high-resolution scanner (dotSlide System, Olympus, Tokyo, Japan) and subdivided into a collection of non-overlapping red, green, and Blue (RGB) images in TIFF format (final resolution, 3.125 pixels/µm). For necrosis quantification, images were acquired at 2× magnification without further subdivision. Image analysis was performed using the open source imaging software ImageJ (http://rsb.info.nih.gov/ij/). Routines for image analysis were coded in ImageJ macro language and executed on RGB images without further treatment. Per each experimental condition, at least three tissue sections from at least three different tumor nodules were analyzed. Necrotic areas was evaluated on TUNEL-stained sections as previously described [27]. Endothelial cells were analyzed on cryosections from in vivo biotinylated mice which were stained with HRP-conjugated streptavidin. Automatic routines were validated by comparing results with those obtained by visual counting of up to 10% of the total images.

### Confocal microscopy

Confocal microscopy was performed as previously described [27]. To detect tumor vessels, frozen sections were incubated with Alexa Fluor 488-conjugated streptavidin. To detect pericytes and tumor vessels, frozen sections were double stained with Alexa Fluor 568-conjugated streptavidin and anti-mouse NG2. Formalin-fixed, paraffin-embedded tumor nodules were sectioned at 1 µm and double-stained with anti-mouse CD31 (Santa Cruz Biotechnology, Inc., Heidelberg, Germany, EU) and anti human/mouse phospho-AKT (Ser473) or phospho-ERK1/2 (Cell Signaling). CD31 expression was revealed by appropriate Alexa Fluor 568-conjugated secondary antibody (Invitrogen) and tumor/endothelial p-AKT or p-ERK1/2 expression was revealed by Alexa Fluor 488- conjugated secondary antibody (Invitrogen). Nuclei were detected incubating sections with TO-PRO-3 nuclear dye (Invitrogen). Fluorescence-stained sections were examined under an epifluorescent microscope equipped with a laser confocal system (MRC-1024, Bio-Rad Laboratories). Image processing was carried out with LaserSharp computer software (Bio-Rad Laboratories).

### Statistical analysis

Statistical analysis was performed with the statistical package Prism 5 (GraphPad Software, San Diego, CA, USA) run on a Macintosh Pro personal computer (Apple Computer Inc.). To test the probability of significant differences between untreated and treated samples, the Student's t test for unpaired data (two-tailed) or two-way ANOVA were used, as appropriate. Tumor volume data and proteome profiler array data were statistically analyzed with two-way ANOVA, and individual group comparisons were evaluated by Bonferroni's multiple comparison test. Differences were considered significant if *p*≤0.05.

## Results

### Sorafenib reduces viable cell countings and triggers apoptosis

**Figure 1:**
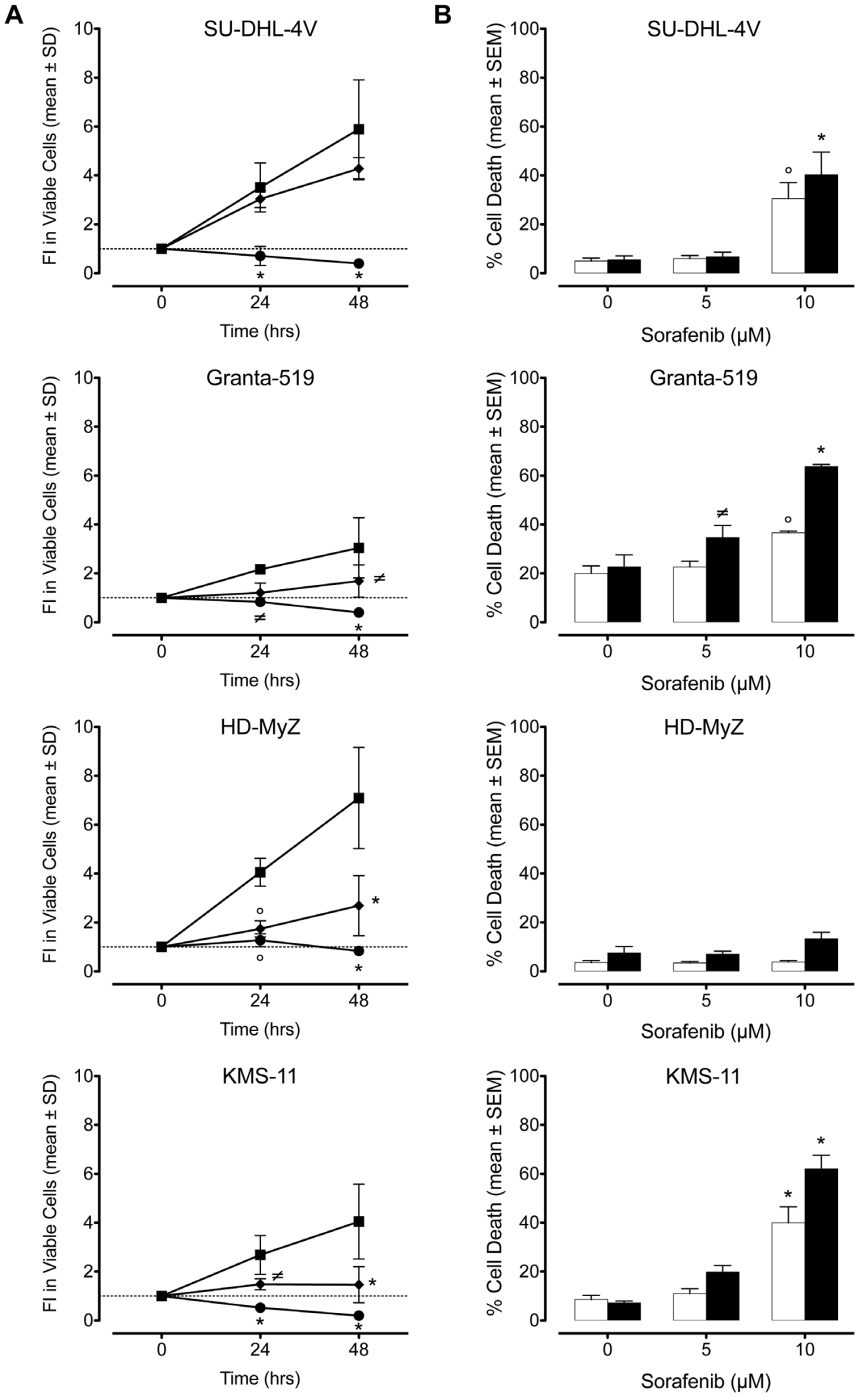
Antiproliferative and apoptotic effects of sorafenib. Following incubation with increasing doses of sorafenib, viable cell counts and apoptotic cell death were assessed by annexin-V/PI double staining and flow cytometry analysis. (**A**) Viable cell counts upon exposure to 0 (▪), 5 (□) and 10 (•) µM sorafenib. Viable cells are expressed as fold increase (FI) of annexin-V^−^/PI^−^ cells after 24 and 48 hours of incubation with sorafenib as compared to day 0. (**B**) Cell death following 24 (□) or 48 (▪) hours sorafenib exposure. Percentages of cell death include annexin-V^+^/PI^−^ plus annexin-V^+^/PI^+^ plus annexin-V^−^/PI^+^ cells. Values refer to four independent experiments. * *p*≤0.001, ° *p*≤0.01, and ≠ *p*≤0.05, compared to controls.

The effects of sorafenib on cell proliferation were investigated in vitro by performing dose-response and time-course experiments using four cell lines representative of lymphoproliferative disorders with different histotypes. Readout of these experiments included viable cell countings and a WST-based cell proliferation assay. Exposure to 5 µM sorafenib for 24 or 48 hours resulted in modest cytostatic effects (**Figure 1A** **, Figure S1**). In contrast, in vitro exposure to a clinically achievable concentration of sorafenib (10 µM) [30] for 24–48 hours resulted in significant reductions of cell viability (**Figure 1B** **, Figure S1**) (*p*≤0.05 at least) with cytotoxic effects being detected in SU-DHL-4V, Granta-519, and KMS-11 cell lines and cytostatic effects being detected in HD-MyZ cell line (**Figure 1B**).

**Figure 2:**
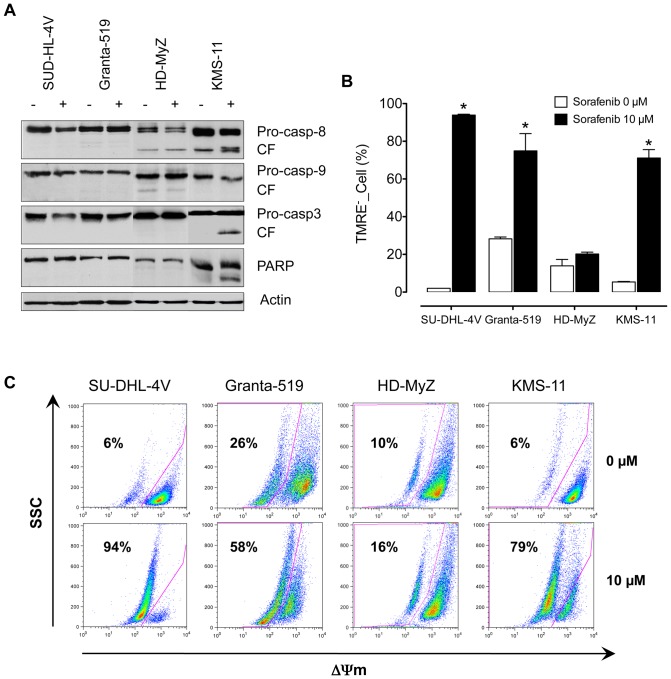
Mechanism of sorafenib-induced apoptosis. SU-DHL-4V, Granta-519, HD-MyZ and KMS-11 cells were treated with sorafenib (10 µM) for 48 hours. (**A**) Cytosolic proteins were then separated by SDS-PAGE and analyzed by immunoblotting with anti-caspases-8, -9, -3, and anti-PARP. CF, indicates cleaved fragments. (**B**) Loss of mitochondrial potential was measured using TMRE staining and flow cytometry. * *p*≤0.001, compared to controls. (**C**) Representative dot plots of mitochondrial membrane depolarization in untreated and sorafenib-treated cell lines.

To investigate whether sorafenib-induced antiproliferative effects involved apoptosis, annexin-V/PI double staining was performed (**Figure 1B** **, Figure S2**). Again, exposure to 5 µM sorafenib had no effect on cell death, whereas exposure to 10 µM sorafenib significantly increased (*p*≤0.05 at least) cell death in all cell lines but HD-MyZ cells. Sensitive cell lines showed a wide degree of variability to sorafenib-induced apoptosis, with mean cell death values of 35% (range, 30% to 40%) and 55% (range, 40% to 64%) following a 24- and 48-hour incubation, respectively (**Figure 1B**). Upon sorafenib exposure, SU-DHL-4V and Granta-519 cell lines but not KMS-11 cell line showed evidences of caspase-independent apoptosis as suggested by the lack of caspase activation and PARP cleavage (**Figure 2A**). To further examine the molecular mechanisms whereby sorafenib triggers apoptosis, we investigated whether loss of mitochondrial potential was involved in sorafenib-induced cell death. Indeed, a potent mitochondrial membrane depolarization could be detected in all apoptosis-prone cell lines (**Figures 2B–C**), with loss of mitochondrial potential ranging from 58% to 94%.

### Effects of sorafenib on primary DLBCL and MCL cells

Apoptotic activity of sorafenib was further evaluated by analyzing its effect on primary cells purified from the peripheral blood of DLBCL and MCL patients at diagnosis (**Table 1**). While sorafenib-induced apoptosis could be detected in only 1 out of 3 MCL samples, apoptotic cell death was consistently observed in all DLBCL samples.

**Table 1:** Effects of sorafenib on primary DLBCL and MCL cells.

Case	Diagnosis	Annexin-V-/PI- (%)		
		DMSO	5 µM	10 µM
1	DLBCL	100	100	59
2	DLBCL	100	100	79
3	DLBCL	100	100	56
4	MCL	100	100	49
5	MCL	100	100	100
6	MCL	100	100	100

Freshly isolated blood MNCs from DLBCL and MCL patients were resuspended (1×10^6^/ml) in RPMI-1640 supplemented with FBS (10%, v/v) and incubated (24 hours, 37°C, 5% CO_2_) with sorafenib (5–10 µM). Controls were supplemented with DMSO. At the end of the incubation, viable cell counts (Annexin-V-/PI-) were evaluated by Flow-Count beads and Annexin-V/PI double staining. Data are expressed as percent of control.

### Sorafenib affects phosphorylation of MAPK and PI3K/Akt pathways

**Figure 3:**
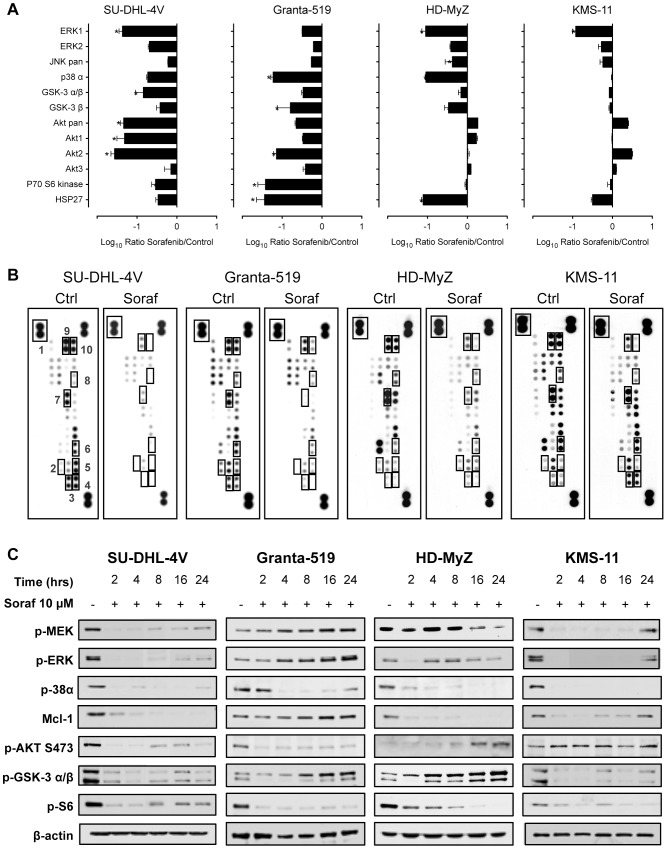
Sorafenib treatment induced changes in survival signals in NHL cells. (**A**) To assess the effects of sorafenib, cells were exposed for 2 hours to control vehicle (DMSO) or sorafenib (10 µM). Shown are data from one of two independent experiments. The chemiluminescence signal intensity of individual spots was analyzed using the open source imaging software ImageJ (http://rsb.info.nih.gov/ij/). The assays were conducted in duplicate. A ratio of signal intensity (sorafenib∶control) was calculated for each of the four replicates (two duplicates per assay) and transformed into a log value (base 10). * *p*≤0.001, and ° *p*≤0.01, compared to controls. (**B**) Shown are data from one of two independent experiments. Representative proteome profiles of control and sorafenib-treated cell lines. The position of the antibodies (double spots for each antibody) relative to the relevant protein kinases is shown. The chemiluminescence signal intensity of individual spots was analyzed using the open source imaging software ImageJ (http://rsb.info.nih.gov/ij/). 1 = positive control; 2 = p70 S6 kinase; 3 = Akt pan; 4 = Akt 2; 5 = Akt 1; 6 = GSK-3 α/β; 7 = p38 α; 8 = JNK pan; 9 = ERK2; and 10 = ERK1. (**C**) Immunoblots of extracts from SU-DHL-4V, Granta-519, HD-MyZ and KMS-11 cells treated with control vehicle (DMSO) or sorafenib (10 µM) for the indicated time periods showed consistent downregulation of p-38α phospho-ERK, phospho-MEK, phospho-Akt, phospho-S6, phospho- GSK-3 α/β and Mcl-1. Equal protein loading was confirmed by blotting for β-actin.

To analyze sorafenib's effects on MAPK and PI3K/Akt signaling pathways, we used a standard antibody array technology allowing simultaneous analysis of all three major MAPKs, including ERK1/ERK2, JNKs, and p38α, as well as the Akt isoforms, and the direct and indirect Akt targets GSK-3 α/β and p70 S6 kinase 1 (p70S6K). Sorafenib (10 µM, 2 hours) affected MAPK and PI3K/Akt signaling pathways in a rather heterogeneous manner (**Figure 3A–C**). As demonstrated by reduced phosphorylation levels for ERK1/2 and p38α kinases, sorafenib downregulated MAPK signaling in all cell lines. Analysis of PI3K/Akt signaling revealed significantly reduced phosphorylation levels of Akt1 and Akt2 in SU-DHL-4V and Granta-519 cell lines. These lines also showed reduced phosphorylation levels of the direct and indirect Akt targets GSK-3 α/β and p70S6K, revealing that sorafenib may severely affect the PI3K/Akt pathway in some lymphoma cell lines (**Figure 3A–B**). In contrast, PI3K/Akt signaling was unaffected in the HD-MyZ, and KMS-11 cell lines.

Western blot analysis essentially confirmed the previous antibody array results, further showing that sorafenib treatment modulated MAPK and PI3K/Akt phosphorylation levels in a rather heterogeneous manner depending on the different target cells (**Figure 3C**). Consistent with sorafenib's effect on the Raf/MEK/ERK pathway, a time-dependent down regulation of MEK and ERK phosphorylation could be detected in SU-DHL-4V, HD-MyZ, and KMS-11 cell lines (**Figure 3C**). Additionally, exposure to sorafenib resulted in a time-dependent down regulation of p-38α in all cell lines. SU-DHL-4V and Granta-519 cells demonstrated constitutive phosphorylation of Akt, which was completely inhibited by sorafenib in a time-dependent manner. Phosphorylation of S6, downstream target proteins of Akt, was also markedly inhibited in all cell lines (**Figure 3C**). Upon sorafenib exposure, expression of Mcl-1 was markedly down regulated in SU-DHL-4V, HD-MyZ and KMS-11 cells, whereas no effect was observed in Granta-519 cells (**Figure 3C**).

### Sorafenib inhibits tumor cell proliferation and angiogenesis

**Figure 4:**
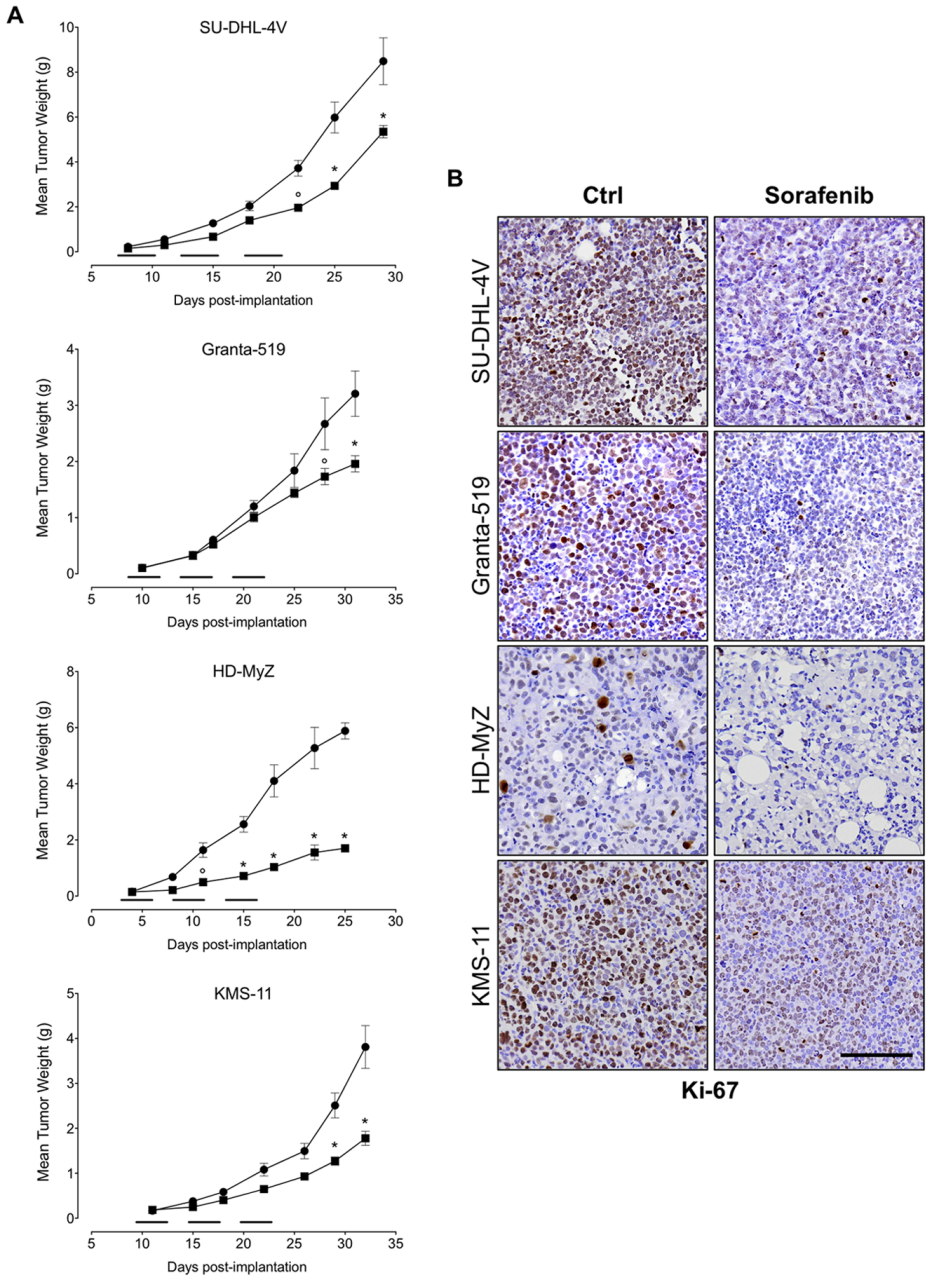
Effect of sorafenib on tumor growth. (**A**) NOD/SCID mice bearing SC tumor nodules 100 mg in weight were randomly assigned to receive a 15-day treatment with sorafenib (▪) (90 mg/kg/day, 5 days per week over 3 weeks) or control vehicle (DMSO) (•). Mice were checked twice weekly for tumor appearance, tumor dimensions, body weight, and toxicity. Mean (± SEM) values refer to at least two independent experiments, using 5 mice per experiment. Treatment initiation is indicated by horizontal black lines. * *p*≤0.001 and ° *p*≤0.01 compared to controls. (**B**) Ki-67 staining of lymphoma tumor treated with sorafenib (90 mg/kg/day, 5 days) or control vehicle (DMSO). In the Ki-67-stained section, brown staining represents positive signals within the tumors (blue cells are the negative, living cells). Objective lens, original magnification: 0.75 NA dry objective, 20×. Scale bar: 50 µm.

NOD/SCID mice inoculated subcutaneously with SU-DHL-4V, Granta-519, HD-MyZ, or KMS-11 cells, received a 15-day treatment with sorafenib (90 mg/kg/day, 5 days per week for 3 weeks) or vehicle control. Treatment was started when tumor volumes reached approximately 100 mg in weight (i.e., 6–13 days after tumor inoculation). Sorafenib treatment resulted in modest effects on the growth of SU-DHL-4V, Granta-519, and KMS-11 xenografts, as shown by TGI values of 37%, 39%, and 53%, respectively, whereas it substantially affected the growth of HD-MyZ xenograft, resulting in a 71% TGI (**Figure 4A**). These findings were paralleled by a strong decrease of Ki-67 expression in tumor cells (**Figure 4B**), suggesting that sorafenib indeed inhibited tumor cell proliferation. However, such an antiproliferative effect was not associated with an increase of tumor cell apotosis, as evaluated by TUNEL staining (**data not shown**), suggesting that the effect of sorafenib on tumor cell growth was cytostatic rather than cytotoxic.

**Figure 5:**
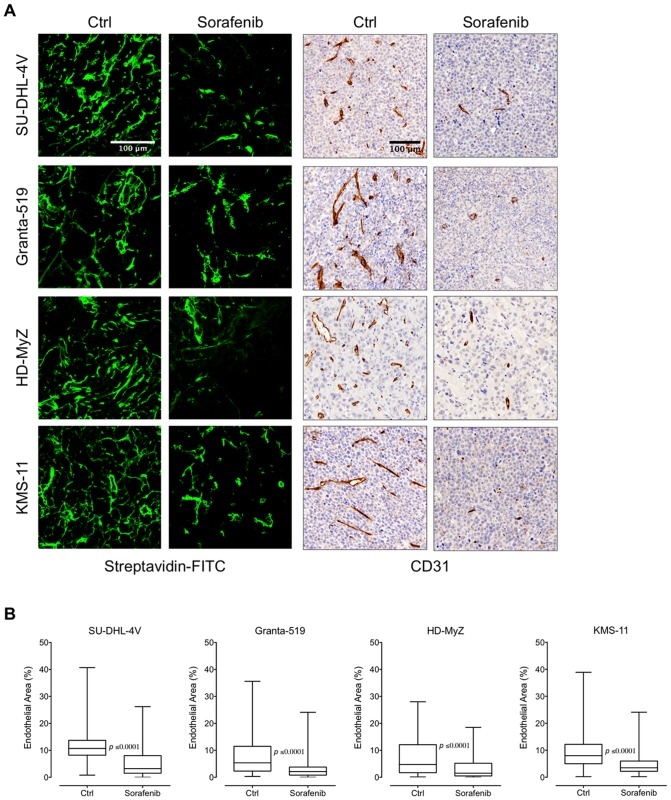
Effect of sorafenib on tumor vasculature. (**A**) Mice treated with sorafenib (90 mg/kg) or control vehicle (DMSO) were in vivo biotinylated with sulfo-NHS-LC-biotin, and tumor vasculature was revealed by staining sections with Alexa Fluor 488-streptavidin (upper panel, objective lens, original magnification: 1.0 NA oil objective, 40×). CD31-stained tumor paraffin sections are shown for comparison (lower panel, objective lens, original magnification: 0.75 NA dry objective, 20×). (**B**) Quantification of endothelial area on entire tissue sections was achieved with ImageJ software. The reduction of vessel density detected in sorafenib-treated nodules was calculated by assessing only viable areas of tissue sections while excluding necrotic areas. The boxes extend from the 25^th^ to the 75^th^ percentiles, the lines indicate the median values, and the whiskers indicate the range of values.

We then evaluated the effects of sorafenib on tumor vasculature by using a sulfo-NHS-LC-biotin-based in vivo assay that allows a detailed qualitative and quantitative analysis of tumor vasculature [29]. Tumor vessel density was assessed by quantifying the percentage of biotinylated cells, i.e., the percentage of the entire tissue section occupied by endothelium. Reduction of tumor vasculature was calculated within viable tumor areas only while excluding necrotic areas. Regardless of the lymphoma subtype, tumor vasculature in control mice was abundant, tortuous, and evenly distributed throughout the tumor, which thus appeared well vascularized (**Figure 5A**). Extensive vascularization is likely due to the upregulation of VEGFR-2 expression by tumor cells and tumor endothelium and the concomitant production of VEGF by tumor cells (data not shown) [31].

In control mice, vascular density resulted in mean values of endothelial areas ranging from 7% to 11% of the total tumor area. Upon a 5-day sorafenib treatment, all tumors showed an average 50% inhibition of tumor vessel density (**Figure 5A**). In fact, as compared to mice receiving vehicle control, sorafenib significantly reduced mean endothelial areas of SU-DHL-4V (11% vs 6%, *p*≤0.0001), Granta-519 (8% vs 3%, *p*≤0.0001), HD-MyZ (7% vs 3%, *p*≤0.0001), and KMS-11 (9% vs 5%, *p*≤0.0001) nodules (**Figure 5B**). The potent sorafenib-induced inhibition of tumor vascularization was further confirmed by staining tumor sections with the endothelial cell marker CD31 (**Figure 5A**).

**Figure 6:**
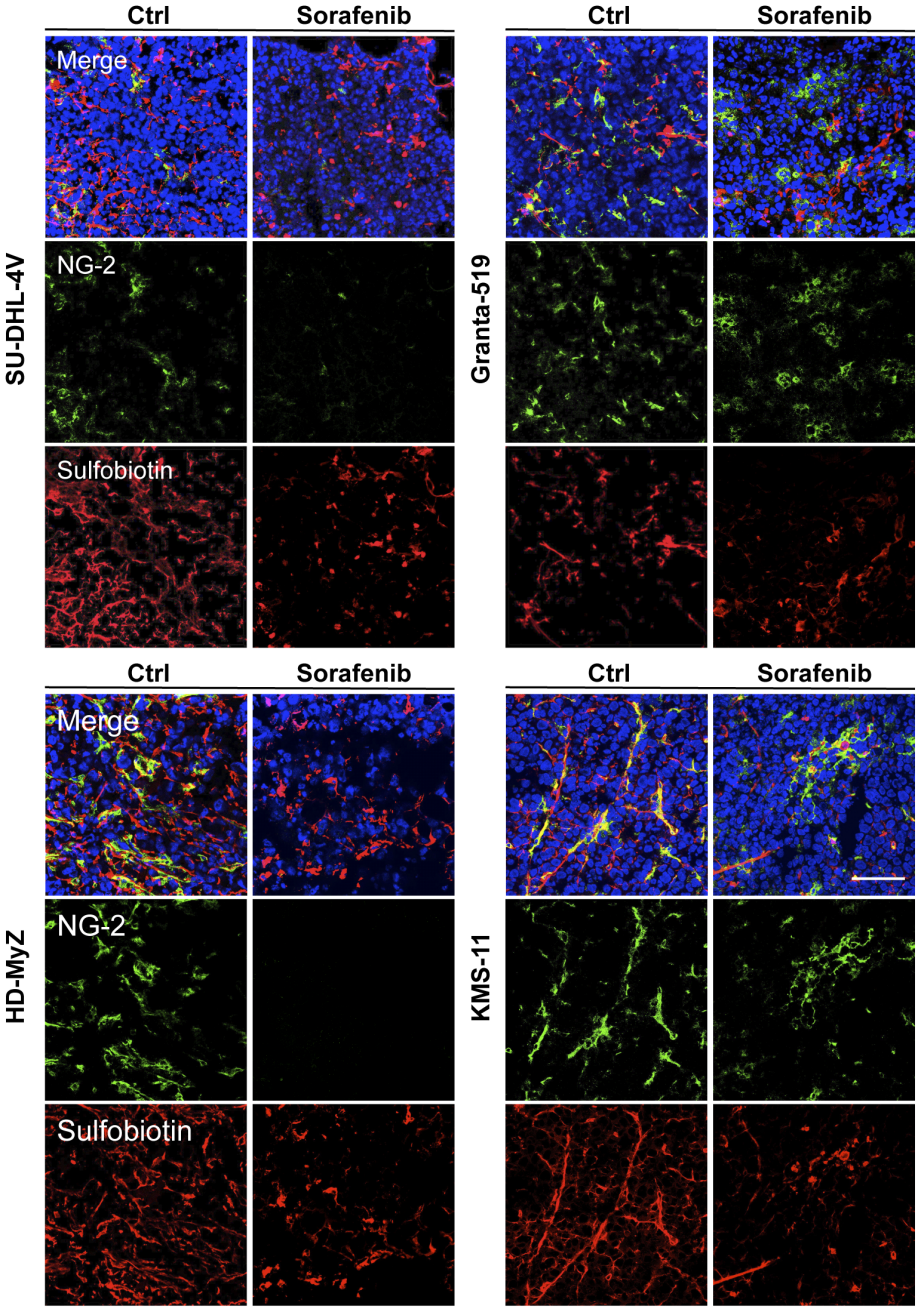
Effects of sorafenib on pericytes. Mice treated with sorafenib (90 mg/kg/die, 5 days) or control vehicle (DMSO) were in vivo biotinylated with sulfo-NHS-LC-biotin. SU-DHL-4V, Granta-519, HD-MyZ and KMS-11 tumor vasculature was revealed by staining sections with Alexa Fluor 568-streptavidin (red). Tumor sections were stained with NG-2 (green) followed by AlexaFluor 488-conjugated secondary antibody for indirect immunofluorescent detection of pericytes. Nuclei were detected with DAPI nuclear dye (blue). Representative images are shown. Objective lens, original magnification: 1.0 NA oil objective, 40×. Scale bar: 100 µm.

In sorafenib-treated tumors, vessels appeared smaller in length, lacking in sproutings and much less arborized and dishomogeneously distributed throughout tumor tissue as compared to vehicle-treated animals (**Figure 5A**) [32], suggesting that in contrast with other antiangiogenic drugs, sorafenib has no effect on vessel normalization [33]. To further investigate this issue, tumor sections were co-stained for both tumor vasculature with Alexa Fluor 568-conjugated streptavidin, and pericytes with anti-NG2 antibody. After sorafenib treatment, confocal microscopy analysis showed a marked reduction of pericytes in SU-DHL-4V and HD-MyZ xenografts, whereas no difference was observed in Granta-519 and HD-MyZ xenografts, further supporting no activity of sorafenib on vessel normalization (**Figure 6**).

**Figure 7:**
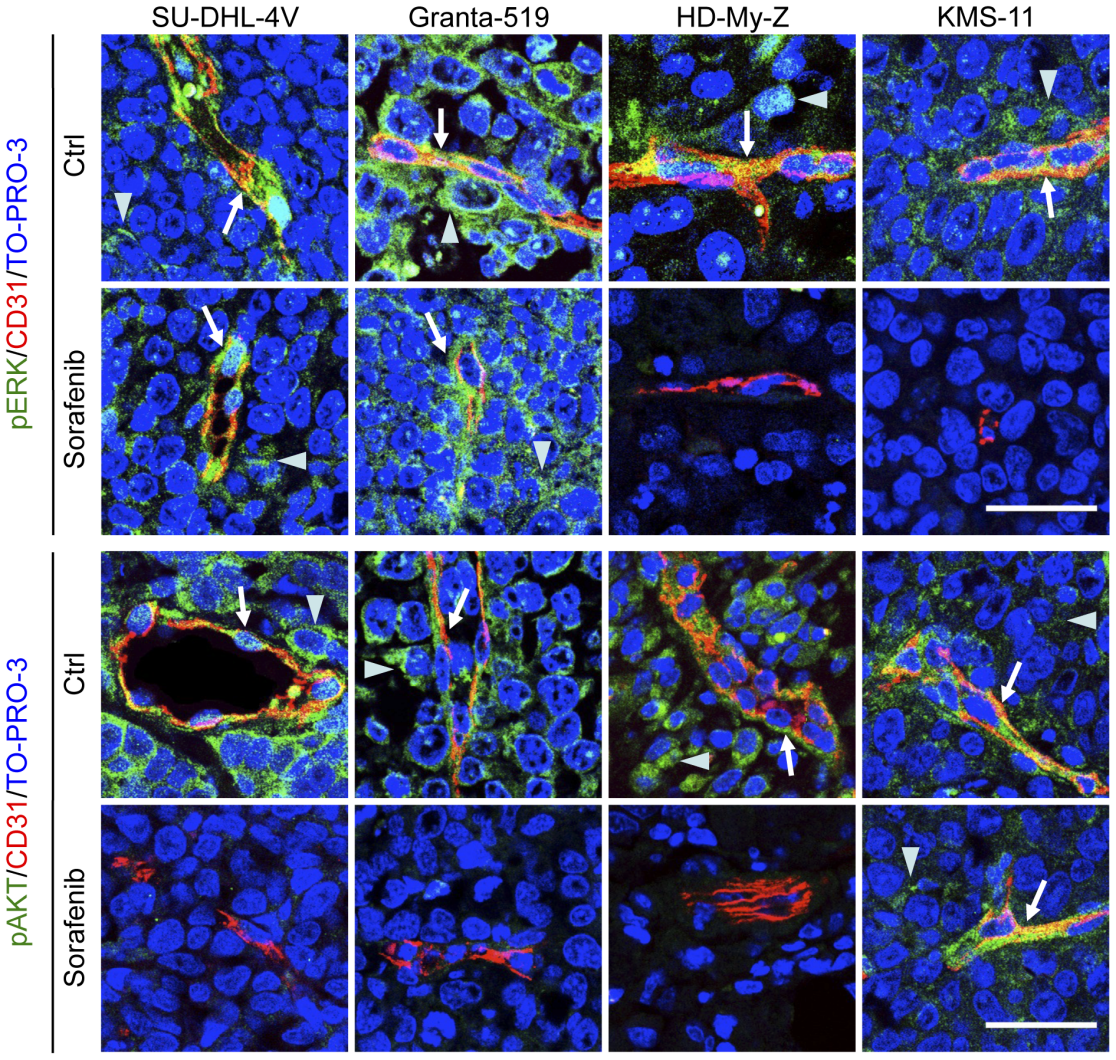
Sorafenib-induced inhibition of Akt and ERK phosphorylation in tumor and endothelial cells. SU-DHL-4V, Granta-519, HD-MyZ and KMS-11 tumor nodules growing subcutaneously in mice treated with sorafenib (90 mg/kg) or control vehicle (DMSO) for 5 days were excised 3 hours after the last treatment, fixed in formalin and embedded in paraffin. Tumor sections were double-stained with CD31 (red) and phospho-ERK 1/2 (green) or phospho-Akt (green) followed by the appropriate AlexaFluor 568- or 488-conjugated secondary antibody for indirect immunofluorescent detection of the corresponding antigen. Nuclei were detected with TO-PRO-3 nuclear dye (blue). Arrows indicate phospho-ERK 1/2 or pospho-Akt expression by endothelial cells; arrowheads indicate phospho-ERK 1/2 or pospho-Akt expression by tumor cells. Representative images are shown. Objective lens, original magnification: 1.0 NA oil objective, 40×. Scale bar: 50 µm.

In vivo anti-tumor mechanism(s) of sorafenib were additionally investigated by analyzing Akt and ERK1/2 phosphorylation in sorafenib-treated tumor cells and tumor vasculature (**Figure 7**). Tumor and vascular ERK1/2 phosphorylation was unaffected in SU-DHL-4V and Granta-519 cell lines, whereas it was strongly reduced in HD-MyZ and KMS-11 cell lines. Tumor and vascular Akt phosphorylation was reduced in SU-DHL-4V, Granta-519 and HD-MyZ cell lines, but not in KMS-11 cell line. Thus, HD-MyZ xenograft showed a combined inhibition of ERK1/2 and Akt phosphorylation on both tumor and endothelial cells, whereas the remaining lymphoma xenografts showed a selective inhibition of either ERK1/2 or Akt pathways on both tumor and vascular cells (**Figure 7**). Notwithstanding the strong inhibition of ERK1/2 and/or Akt phosphorylation on vascular cells, we did not detect any increase in tumor endothelial cell apoptosis, as evaluated by CD31/TUNEL double-staining (data not shown).

**Figure 8:**
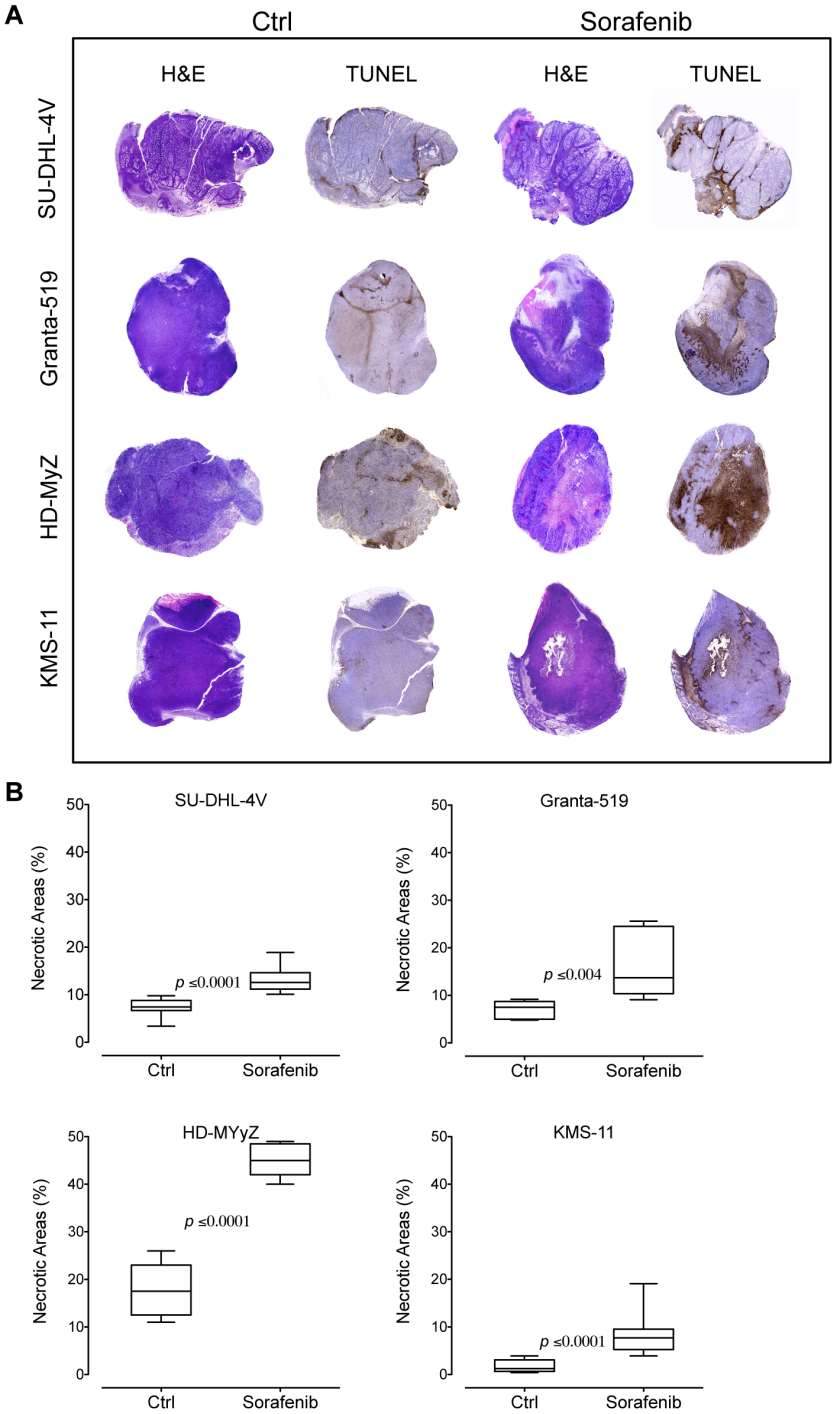
Sorafenib induces in vivo tumor necrosis. (**A**) H&E and TUNEL staining of lymphoma tumor treated with sorafenib (90 mg/kg) or control vehicle (DMSO). In the TUNEL-stained section, brown staining represents positive signals within the tumors (blue cells are the negative, living cells). Objective lens, original magnification: 0.08 NA dry objective, 2×. (**B**) Quantification of necrotic areas on entire tissue sections using ImageJ software. Percentage of necrosis was calculated by the following formula: (necrotic area/total tissue area)×100. The boxes extend from the 25^th^ to the 75^th^ percentiles, the lines indicate the median values, and the whiskers indicate the range of values.

Despite apoptosis was not a prominent features of tumor or endothelial cells in sorafenib-treated mice, tumor sections from these animals showed large areas of non-hemorrhagic tumor necrosis by hematoxilyn/eosin as well as TUNEL staining (**Figure 8A**), suggesting that hypoxic conditions subsequent to tumor vessel inhibition might have triggered tumor destruction. As compared to controls, sorafenib significantly increased necrotic areas in mice bearing SU-DHL-4V (7% vs 13%, *p*≤0.0001), Granta-519 (7% vs 17%, *p*≤0.004), HD-MyZ (18% vs 45%, *p*≤0.0001), and KMS-11 (2% vs 8%, *p*≤0.0001) xenografts (**Figure 8B**).

## Discussion

Data reported herein using four cell lines representative of different lymphoproliferative disorders show that sorafenib may exert antilymphoma activities that are likely mediated by a variety of mechanism(s), including antiproliferative effects on tumor cells as well as antiangiogenic effects. These findings significantly extend the spectrum of tumors that sorafenib targets and are consistent with previously reported results showing efficacy of sorafenib in multiple tumor xenograft models [7], [15]–[17].

In vitro, clinically achievable concentrations of sorafenib (5–10 µM) [30] induced a marked inhibition of MAPK and/or PI3K/Akt signaling as well as Mcl-1 in all cell lines, resulting in a significant reduction of cell proliferation and/or induction of apoptosis. Sorafenib affected both MAPK and PI3K/Akt signaling in essentially all analyzed cell lines, with PI3K/Akt signaling being markedly affected in SU-DHL-4V and Granta-519 cell line. In vivo, sorafenib has previously been reported to inhibit the growth of a wide variety of human tumor xenografts in mice [7], [15]–[17]. Our data obtained in four xenograft models of lymphoma demonstrate that the potent antiangiogenic activity of sorafenib, eventually combined with its marked inhibition of lymphoma cell proliferation, induces a relevant tumor destruction as shown by large areas of tumor necrosis detected in sorafenib-treated mice. According to previously reported studies, an optimal biological dose of sorafenib (90 mg/kg/day) was choosen for our in vivo experiments [9], [18], [34]. Sorafenib dose resulting in antitumor activity in our xenograft models (90 mg/kg/day) roughly equals to 600 mg/day in humans and compares well with the standard sorafenib daily dose used in clinical trials. The treatment dose used in our experiments is expected to produce plasma drug concentrations in the range of pharmacologically achievable concentrations of sorafenib, i.e., 5 to 15 µM, that are consistently detected in patients receiving the standard sorafenib daily dose of 800 mg [4], [5], [17], [30].

While a 5-day treatment with sorafenib induced an average 50% inhibition of tumor angiogenesis and extensive tumor necrosis, biologically significant effects on the volume of lymphoma xenografts could only be observed in one of four xenografts (i.e., HD-MyZ) and required a long-term sorafenib treatment. Indeed, the limited effects of sorafenib on the volume of lymphoma xenografts closely resemble the findings frequently reported in patients treated with sorafenib who may experience a clinical benefit in the absence of any tumor volume reduction [3], [35].

Analysis of tumor xenografts showed a substantial reduction of Ki-67 expression, as well as inhibition of phospho-Akt and/or phospho-ERK1/2 levels, suggesting that sorafenib-mediated lymphoma growth inhibition also involves a direct inhibition of cancer cell proliferation. However, analysis of tumor nodules from mice receiving a 5-day sorafenib treatment revealed a marked reduction of tumor angiogenesis, eventually associated with inhibition of vascular phospho-Akt and/or phospho-ERK1/2, thus suggesting that inhibition of angiogenesis is likely to play a key role in triggering the antitumor activity of sorafenib. Indeed, angiogenesis inhibition almost certainly represents a primary effect of sorafenib treatment rather than being secondary to tumor cell loss and reduced production of angiogenic factors. In fact, inhibition of tumor angiogenesis was detected in non-necrotic areas, suggesting that inhibition of tumor vasculature was the first event triggered by sorafenib, subsequently leading to the generation of extensive tumor necrosis due to hypoxic conditions. Alternatively, if tumor cell loss would have preceded reduction of angiogenesis, tumor vessel density would not be expected to decrease and should result unchanged or possibly increased. Taken together, these data strongly support that sorafenib acts primarily as an antiangiogenic agent, as shown by the marked tumor vessel reduction, but it also directly affects lymphoma cells, as shown by Ki-67 inhibition.

It has been proposed that one of the mechanisms by which antiangiogenic agents mediate their effect is to transiently “normalize” tumor vasculature, resulting in attenuation of hyperpermeability, increased vascular pericyte coverage, reduction in tumor hypoxia and interstitial fluid pressure [36]. The results of our studies however, show that administration of sorafenib results in a decrease of vasculature, leading to elevated level of tumor necrosis with the absence of vessel normalization as detected by vessel morphological features as well as number of pericytes. Although vessels leakiness poses a challenge for drug delivery [37], at the same time, lack of pericytes makes the vessels susceptible to antiangiogenic therapies [38]. Sorafenib may therefore improve anti-angiogenic therapy in lymphoma by targeting both endothelial cells as well as their supporting pericytes.

The best in vivo antitumor response to sorafenib, i.e., a 70% TGI, was detected in NOD/SCID mice bearing HD-MyZ xenografts that showed a marked inhibition of both tumor and vascular ERK1/2 and Akt phosphorylation, suggesting that the concomitant inhibition of MAPK and PI3K/Akt signaling is required to achieve an optimal antilymphoma activity. In contrast, modest in vivo responses, ranging from 35% to 50% TGIs, were observed in mice bearing SU-DHL-4V, Granta-519 or KMS-11 xenografts that were characterized by a selective in vivo inhibition of either ERK1/2 or Akt signaling. Overall, MAPK, PI3K/Akt and Mcl-1 signaling pathways appear to be critical determinants mediating the in vivo antitumor response to sorafenib [12], [14], [39].

Human lymphomas harbor multiple genetic and epigenetic alterations leading to deregulation of multiple pathways and cellular processes. Our data as well as those of others [19]–[22] suggest that the therapeutic armamentarium today available for human lymphomas might also include sorafenib mainly due to its antiangiogenic properties along with its antiproliferative and signaling inhibitory activities eventually associated with down modulation of Mcl-1 expression [31], [40], [41]. As a single agent, sorafenib might preferentially act as an inducer of tumor necrosis, whereas it might enhance the efficacy of radio-chemotherapy due to its effects on vessel normalization. In conclusion, our results provide evidence that sorafenib may be a potentially interesting molecule for the treatment of lymphoproliferative disorders mainly due to its antiangiogenic properties.

## Supporting Information

Figure S1 **Cell growth inhibition of sorafenib toward NHL cells.** NHL cells were treated with the indicated concentrations of sorafenib for 24–48 hours. Cell viability was measured using WST assays. * *p*≤0.0001 and ≠ *p*≤0.001 compared to controls.
(TIF)

Figure S2 **Sorafenib cytotoxicity.** Cell death was assessed by annexin-V/PI double staining and flow cytometry analysis. Representative dot plots of cell death in untreated and sorafenib-treated (5–10 µM) cell lines after 48 hours of exposure.
(TIF)

## References

[1] Kane RC, Farrell AT, Saber H, Tang S, Williams G, et al (2006) Sorafenib for the treatment of advanced renal cell carcinoma. Clinical Cancer Research 12: 7271–7278. 17189398 10.1158/1078-0432.CCR-06-1249

[2] Escudier B, Eisen T, Stadler WM, Szczylik C, Oudard S, et al (2007) Sorafenib in advanced clear-cell renal-cell carcinoma. The New England Journal of Medicine 356: 125–134. 17215530 10.1056/NEJMoa060655

[3] Llovet JM, Ricci S, Mazzaferro V, Hilgard P, Gane E, et al (2008) Sorafenib in advanced hepatocellular carcinoma. N Engl J Med 359: 378–390. 18650514 10.1056/NEJMoa0708857

[4] Clark JW, Eder JP, Ryan D, Lathia C, Lenz HJ (2005) Safety and pharmacokinetics of the dual action Raf kinase and vascular endothelial growth factor receptor inhibitor, BAY 43-9006, in patients with advanced, refractory solid tumors. Clinical Cancer Research 11: 5472–5480. 16061863 10.1158/1078-0432.CCR-04-2658

[5] Strumberg D, Richly H, Hilger RA, Schleucher N, Korfee S, et al (2005) Phase I clinical and pharmacokinetic study of the Novel Raf kinase and vascular endothelial growth factor receptor inhibitor BAY 43-9006 in patients with advanced refractory solid tumors. Journal of Clinical Oncology 23: 965–972. 15613696 10.1200/JCO.2005.06.124

[6] Gupta-Abramson V, Troxel AB, Nellore A, Puttaswamy K, Redlinger M, et al (2008) Phase II trial of sorafenib in advanced thyroid cancer. Journal of Clinical Oncology 26: 4714–4719. 18541894 10.1200/JCO.2008.16.3279 PMC2653134

[7] Zhang W, Konopleva M, Shi YX, McQueen T, Harris D, et al (2008) Mutant FLT3: a direct target of sorafenib in acute myelogenous leukemia. Journal of National Cancer Institute 100: 184–198. 10.1093/jnci/djm328 18230792

[8] Wilhelm S, Carter C, Lynch M, Lowinger T, Dumas J, et al (2006) Discovery and development of sorafenib: a multikinase inhibitor for treating cancer. Nature Reviews Drug Discovery 5: 835–844. 17016424 10.1038/nrd2130

[9] Wilhelm SM, Adnane L, Newell P, Villanueva A, Llovet JM, et al (2008) Preclinical overview of sorafenib, a multikinase inhibitor that targets both Raf and VEGF and PDGF receptor tyrosine kinase signaling. Molecular Cancer Therapeutics 7: 3129–3140. 18852116 10.1158/1535-7163.MCT-08-0013 PMC12261297

[10] Fabian MA, Biggs WH 3rd, Treiber DK, Atteridge CE, Azimioara MD, et al (2005) A small molecule-kinase interaction map for clinical kinase inhibitors. Nature Biotechnology 23: 329–336. 10.1038/nbt1068 15711537

[11] Roberts PJ, Der CJ (2007) Targeting the Raf-MEK-ERK mitogen-activated protein kinase cascade for the treatment of cancer. Oncogene 26: 3291–3310. 17496923 10.1038/sj.onc.1210422

[12] Rahmani M, Davis EM, Bauer C, Dent P, Grant S (2005) Apoptosis induced by the kinase inhibitor BAY 43-9006 in human leukemia cells involves down-regulation of Mcl-1 through inhibition of translation. Journal of Biology Chemistry 280: 35217–35227. 10.1074/jbc.M506551200 16109713

[13] Meng XW, Lee SH, Dai H, Loegering D, Yu C, et al (2007) Mcl-1 as a buffer for proapoptotic Bcl-2 family members during TRAIL-induced apoptosis: a mechanistic basis for sorafenib (Bay 43-9006)-induced TRAIL sensitization. Journal of Biological Chemistry 282: 29831–29846. 17698840 10.1074/jbc.M706110200

[14] Yu C, Bruzek LM, Meng XW, Gores GJ, Carter CA, et al (2005) The role of Mcl-1 downregulation in the proapoptotic activity of the multikinase inhibitor BAY 43-9006. Oncogene 24: 6861–6869. 16007148 10.1038/sj.onc.1208841

[15] Wilhelm SM, Carter C, Tang L, Wilkie D, McNabola A, et al (2004) BAY 43-9006 exhibits broad spectrum oral antitumor activity and targets the RAF/MEK/ERK pathway and receptor tyrosine kinases involved in tumor progression and angiogenesis. Cancer Research 64: 7099–7109. 15466206 10.1158/0008-5472.CAN-04-1443

[16] Sharma A, Trivedi NR, Zimmerman MA, Tuveson DA, Smith CD, et al (2005) Mutant V599EB-Raf regulates growth and vascular development of malignant melanoma tumors. Cancer Research 65: 2412–2421. 15781657 10.1158/0008-5472.CAN-04-2423

[17] Chang YS, Adnane J, Trail PA, Levy J, Henderson A, et al (2007) Sorafenib (BAY 43-9006) inhibits tumor growth and vascularization and induces tumor apoptosis and hypoxia in RCC xenograft models. Cancer Chemotherapy and Pharmacology 59: 561–574. 17160391 10.1007/s00280-006-0393-4

[18] Liu L, Cao Y, Chen C, Zhang X, McNabola A, et al (2006) Sorafenib blocks the RAF/MEK/ERK pathway, inhibits tumor angiogenesis, and induces tumor cell apoptosis in hepatocellular carcinoma model PLC/PRF/5. Cancer Research 66: 11851–11858. 17178882 10.1158/0008-5472.CAN-06-1377

[19] Nguyen TK, Jordan N, Friedberg J, Fisher RI, Dent P, et al (2010) Inhibition of MEK/ERK1/2 sensitizes lymphoma cells to sorafenib-induced apoptosis. Leukemia Research 379–386. 20117835 10.1016/j.leukres.2009.07.013 PMC3150480

[20] Chapuy B, Schuelper N, Panse M, Dohm A, Hand E, et al (2011) Multikinase inhibitor sorafenib exerts cytocidal efficacy against Non-Hodgkin lymphomas associated with inhibition of MAPK14 and AKT phosphorylation. British Journal of Haematology 152: 401–412. 21689083 10.1111/j.1365-2141.2010.08526.x

[21] Ullrich K, Wurster KD, Lamprecht B, Kochert K, Engert A, et al (2011) BAY 43-9006/Sorafenib blocks CSF1R activity and induces apoptosis in various classical Hodgkin lymphoma cell lines. Br J Haematol 155: 398–402. 21517818 10.1111/j.1365-2141.2011.08685.x

[22] Ramakrishnan V, Timm M, Haug JL, Kimlinger TK, Halling T, et al (2012) Sorafenib, a multikinase inhibitor, is effective in vitro against non-Hodgkin lymphoma and synergizes with the mTOR inhibitor rapamycin. Am J Hematol 87: 277–283. 22190165 10.1002/ajh.22263 PMC3465673

[23] Huber S, Oelsner M, Decker T, Zum Buschenfelde CM, Wagner M, et al (2011) Sorafenib induces cell death in chronic lymphocytic leukemia by translational downregulation of Mcl-1. Leukemia 25: 838–847. 21293487 10.1038/leu.2011.2

[24] Minn AJ, Rudin CM, Boise LH, Thompson CB (1995) Expression of bcl-xL can confer a multidrug resistance phenotype. Blood 86: 1903–1910. 7655019

[25] Jazirehi AR, Vega MI, Chatterjee D, Goodglick L, Bonavida B (2004) Inhibition of the Raf-MEK1/2-ERK1/2 signaling pathway, Bcl-xL down-regulation, and chemosensitization of non-Hodgkin's lymphoma B cells by Rituximab. Cancer Research 64: 7117–7126. 15466208 10.1158/0008-5472.CAN-03-3500

[26] Cho-Vega JH, Rassidakis GZ, Admirand JH, Oyarzo M, Ramalingam P, et al (2004) MCL-1 expression in B-cell non-Hodgkin's lymphomas. Human Pathology 35: 1095–1100. 15343511 10.1016/j.humpath.2004.04.018

[27] Lavazza C, Carlo-Stella C, Giacomini A, Cleris L, Righi M, et al (2010) Human CD34+ cells engineered to express membrane-bound tumor necrosis factor-related apoptosis-inducing ligand target both tumor cells and tumor vasculature. Blood 115: 2231–2240. 20075160 10.1182/blood-2009-08-239632

[28] Carlo-Stella C, Di Nicola M, Turco MC, Cleris L, Lavazza C, et al (2006) The anti-human leukocyte antigen-DR monoclonal antibody 1D09C3 activates the mitochondrial cell death pathway and exerts a potent antitumor activity in lymphoma-bearing nonobese diabetic/severe combined immunodeficient mice. Cancer Research 66: 1799–1808. 16452241 10.1158/0008-5472.CAN-05-1200

[29] Rybak JN, Ettorre A, Kaissling B, Giavazzi R, Neri D, et al (2005) In vivo protein biotinylation for identification of organ-specific antigens accessible from the vasculature. Nature Methods 2: 291–298. 15782212 10.1038/nmeth745

[30] Hu S, Niu H, Inaba H, Orwick S, Rose C, et al (2011) Activity of the multikinase inhibitor sorafenib in combination with cytarabine in acute myeloid leukemia. Journal of the National Cancer Institute 103: 893–905. 21487100 10.1093/jnci/djr107 PMC3110171

[31] Gratzinger D, Zhao S, Marinelli RJ, Kapp AV, Tibshirani R, et al (2007) Microvessel density and expression of vascular endothelial growth factor and its receptors in diffuse large B-cell lymphoma subtypes. American Journal of Pathology 170: 1362–1369. 17392174 10.2353/ajpath.2007.060901 PMC1829468

[32] Righi M, Giacomini A, Lavazza C, Sia D, Carlo-Stella C, et al (2009) A computational approach to compare microvessel distributions in tumors following antiangiogenic treatments. Laboratory Investigation 89: 1063–1070. 19652644 10.1038/labinvest.2009.76

[33] Bottos A, Martini M, Di Nicolantonio F, Comunanza V, Maione F, et al (2012) Targeting oncogenic serine/threonine-protein kinase BRAF in cancer cells inhibits angiogenesis and abrogates hypoxia. Proc Natl Acad Sci U S A 109: E353–359. 22203991 10.1073/pnas.1105026109 PMC3277561

[34] Liu LP, Ho RL, Chen GG, Lai PB (2012) Sorafenib inhibits hypoxia-inducible factor-1alpha synthesis: implications for antiangiogenic activity in hepatocellular carcinoma. Clin Cancer Res 18: 5662–5671. 22929805 10.1158/1078-0432.CCR-12-0552

[35] Guidetti A, Carlo-Stella C, Locatelli SL, Malorni W, Pierdominici M, et al (2012) Phase II study of sorafenib in patients with relapsed or refractory lymphoma. Br J Haematol 158: 108–119. 22571717 10.1111/j.1365-2141.2012.09139.x

[36] Jain RK (2005) Normalization of tumor vasculature: an emerging concept in antiangiogenic therapy. Science 307: 58–62. 15637262 10.1126/science.1104819

[37] Carmeliet P, Jain RK (2011) Principles and mechanisms of vessel normalization for cancer and other angiogenic diseases. Nat Rev Drug Discov 10: 417–427. 21629292 10.1038/nrd3455

[38] Bergers G, Song S (2005) The role of pericytes in blood-vessel formation and maintenance. Neuro Oncol 7: 452–464. 16212810 10.1215/S1152851705000232 PMC1871727

[39] Grant S (2008) Cotargeting survival signaling pathways in cancer. Journal of Clinical Investigation 4. 10.1172/JCI36898 PMC2518078 18725993

[40] Ganjoo K, Moore A, Orazi A, Sen J, Johnson C, et al (2008) The importance of angiogenesis markers in the outcome of patients with diffuse large B cell lymphoma: a retrospective study of 97 patients. Journal of Cancer Research and Clinical Oncology 134: 381–387. 17694324 10.1007/s00432-007-0294-x PMC12161654

[41] Gratzinger D, Zhao S, Tibshirani R, Hsi E, Hans C, et al (2008) Prognostic significance of VEGF, VEGF receptors, and microvessel density in diffuse large B cell lymphoma treated with anthracycline-based chemotherapy. Laboratory Investigation 88: 38–47. 17998899 10.1038/labinvest.3700697

