# Supplementary figures and images for: Sorafenib Inhibits Lymphoma Xenografts by Targeting MAPK/ERK and AKT Pathways in Tumor and Vascular Cells
Source: PLoS One. 2013 Apr 19;8(4):e61603. doi: 10.1371/journal.pone.0061603 (PMC3631141; doi:10.1371/journal.pone.0061603)

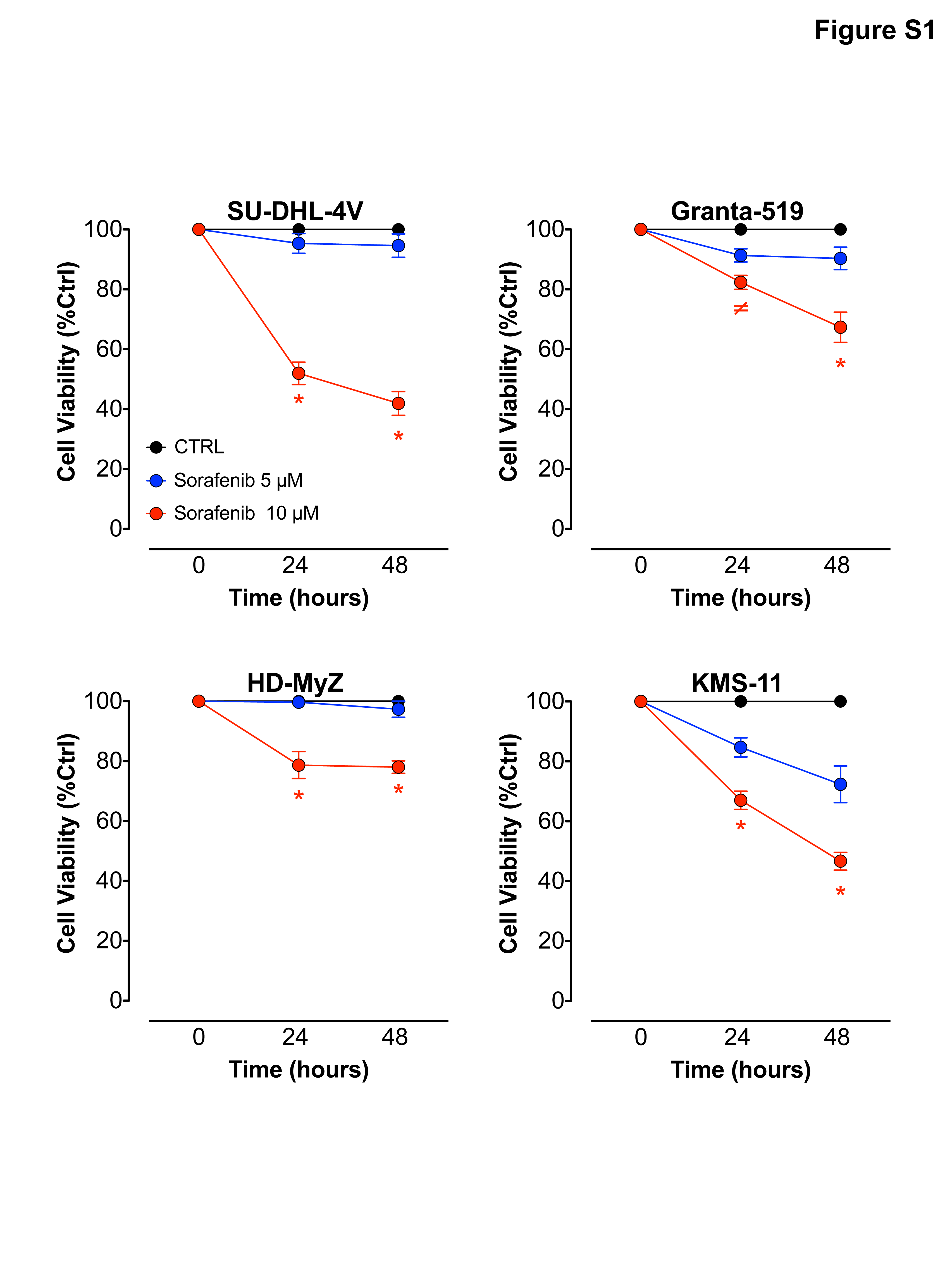

Supplement: Figure S1 — Cell growth inhibition of sorafenib toward NHL cells. NHL cells were treated with the indicated concentrations of sorafenib for 24–48 hours. Cell viability was measured using WST assays. * p≤0.0001 and ≠ p≤0.001 compared to controls. (TIF) [file pone.0061603.s001.tif]

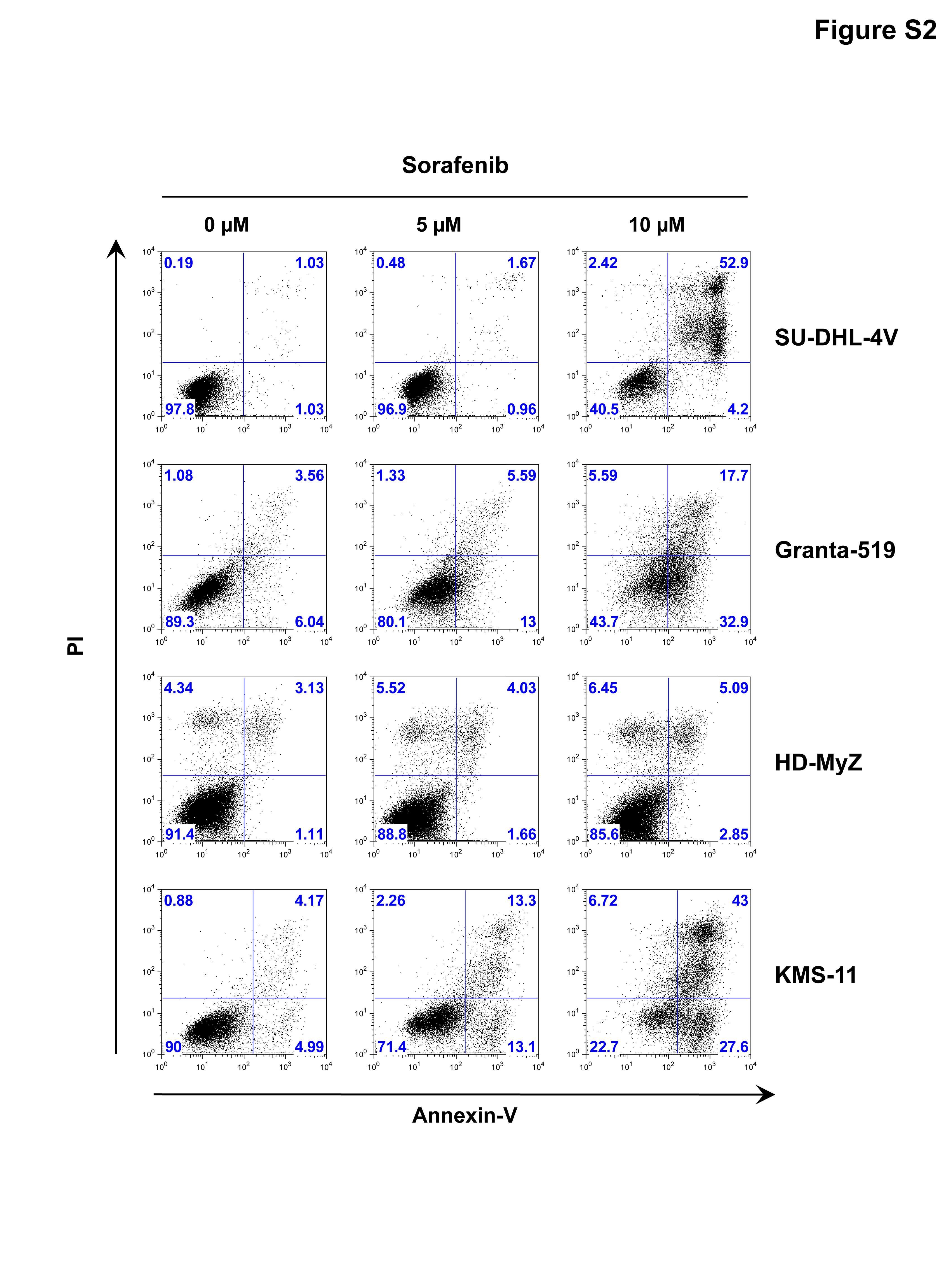

Supplement: Figure S2 — Sorafenib cytotoxicity. Cell death was assessed by annexin-V/PI double staining and flow cytometry analysis. Representative dot plots of cell death in untreated and sorafenib-treated (5–10 µM) cell lines after 48 hours of exposure. (TIF) [file pone.0061603.s002.tif]
